# The effect of parasite infection on the recombination rate of the mosquito *Aedes aegypti*

**DOI:** 10.1371/journal.pone.0203481

**Published:** 2018-10-09

**Authors:** Giacomo Zilio, Lea Moesch, Nathalie Bovet, Anouk Sarr, Jacob C. Koella

**Affiliations:** 1 Institute of Biology, University of Neuchâtel, Neuchâtel, Switzerland; 2 Department of Environmental Systems Science, ETHZ, Zurich, Switzerland; University of Ostrava, CZECH REPUBLIC

## Abstract

Sexual reproduction and meiotic recombination generate new genetic combinations and may thereby help an individual infected by a parasite to protect its offspring from being infected. While this idea is often used to understand the evolutionary forces underlying the maintenance of sex and recombination, it also suggests that infected individuals should increase plastically their rate of recombination. We tested the latter idea with the mosquito Aedes aegypti and asked whether females infected by the microsporidian Vavraia culicis were more likely to have recombinant offspring than uninfected females. To measure the rate of recombination over a chromosome we analysed combinations of microsatellites on chromosome 3 in infected and uninfected females, in the (uninfected) males they copulated with and in their offspring. As predicted, the infected females were more likely to have recombinant offspring than the uninfected ones. These results show the ability of a female to diversify her offspring in response to parasitic infection by plastically increasing her recombination rate.

## Introduction

Genetic recombination shuffles the genes of adults and generate novel genotypes. This has the disadvantage of breaking up genetic associations built by selection and combinations of genes that were beneficial to the adults, replacing them with new ones [1–3]. Nevertheless, the modification of genotypes at each generation through recombination may help the host to respond and adapt to a changing environment [4, 5], in accordance with the abandon-ship hypothesis predicting that, under stressful and poor conditions, sex should be favoured [6–9]. Part of this environmental variation is represented by the biotic pressure imposed by harmful and virulent parasite (i.e. parasites reducing host’s fitness) [10]. Interactions between hosts and parasites are therefore at the basis of several ideas about the maintenance of sexual reproduction [11–13].

It is, for example, often the case that hosts and parasites are genetically variable in their resistance and infectivity [14, 15]. Offspring that are genetically similar to their mother will be more susceptible to an infection by the parasite that was able to overcome the mother’s defences than offspring with more different genotypes. Thus, from the parasite’s point of view, a parasite that has infected a host will be less able to infect that individual’s offspring, if genetic recombination has led to sufficiently large differences between the infected individual and its offspring [16]. Thus, selection will favour offspring that are genetically different from their mother [17].

Since hosts gain an advantage by producing offspring with rare and unusual genetic combinations, selection will tend to maintain a high level of recombination in populations where parasites are common [18, 19]. This is supported, for example, by experiments with the flour beetle *Tribolium castaneum* and the microsporidian *Nosema whitei* [20–22]. The flour beetle reproduces strictly sexually, but its rate of recombination can vary. After experimental populations of the beetle had been allowed to evolve for 11 generations, the frequency of recombination was greater in lines that had coevolved together with *Nosema whitei* than in the parasite-free ones.

Alternatively, changes in the rate of recombination may occur not only in response to evolutionary processes as coevolution with parasites [20–22], but also plastically [23]. Indeed, organisms can respond to stressful conditions and modify their recombination rate according to the environment (phenotypic plasticity). It was, for example, observed more than 100 years ago [24] that the rate of recombination of *Drosophila melanogaster* differs according to temperature, humidity, diet, age, and social status (reviewed in [23]). Such plasticity in the rate of recombination is also found in other animals such as nematodes or grasshoppers, in plants such as tomatoes and tobacco, and in humans [15–34]. However, the phenotypic plasticity of recombination rate in response to parasites is rarely considered. While the few studies in plants [35–37] all found that individuals infected by viruses or with an oomycete have a higher rate of recombination than uninfected ones, the two existing studies in animals gave conflicting results [38, 39]. In response to either bacteria or parasitic wasps, individuals of *Drosophila melanogaster* increase the recombinant fraction of their descendants by transmission distortion of the recombinant chromatids [38], whereas recombination in house mice is not affected by bacterial infection [39].

The aim of our study was to provide further evidence for a plastic increase of the rate of recombination in response to parasitic infection. We therefore asked how the recombination rate of the mosquito *Aedes aegypti* changes if it is infected by the virulent microsporidian parasite *Vavraia culicis*.

## Materials and methods

### Experimental system

We used the UGAL strain of the mosquito *Aedes aegypti*, which was provided by P. Guérin (University of Neuchâtel), and its microsporidian parasite *Vavraia culicis*, which was provided by J. J. Becnel (USDA, Gainesville, USA). *Aedes aegypti* is the main vector of yellow fever, dengue, chikungunya and Zika viruses [40]. Due to the impact on human health its physiology, genetics, and ecology are well known [41, 42]. It is ubiquitous in the tropics and subtropics, where the larvae growing in natural or artificial containers encounter periods of nutrient restriction and competition [43, 44]. The eggs resist desiccation and can be stored for several months. After a single mating the females lay eggs throughout their lifespan [45, 46]. *Vavraia culicis* is an obligate endocellular parasite of several genera of mosquitoes [47], showing a condition-dependent virulence when infecting *Ae*. *aegypti* [48]. Infection occurs in the aquatic environment when the mosquito larvae ingest the spores with their food. The parasite penetrates the gut and epithelial cells, undergoes a series of developmental stages and finally produces the infectious spores. These are usually transmitted horizontally when larvae or pupae die in the water. If infected mosquitoes survive to adulthood, the spores can adhere to the surface of the eggs and infect newly hatched larvae [49] or the mosquitoes dying on the surface of a larval site can release the spores.

### Experimental design

The purpose of this study was to compare the recombination rate of infected and uninfected females. To measure the rate of recombination, we genotyped microsatellites of mothers, fathers and their offspring, and measured for each pair of microsatellites the proportion of offspring that were recombinant. Throughout the experiment, the mosquitoes were maintained at 26°C, 70% humidity, 12 h:12 h light:dark photoperiod.

The experiment was performed in 2 blocks with the same experimental procedure except for the number of microsatellites analysed (Table 1). In the first block we used four microsatellites (A, B, D and F); in the second we used two additional ones (C and E) (for further information see [50]: Additional file 1). We chose microsatellites that are located on the same chromosome (chromosome three), for the markers on different chromosomes can be expected to recombine freely, leading to a recombination fraction that is not affected by the environment. The forward primers of each microsatellite were modified with a commercial fluorochrome (Table 1) for genotyping by capillary electrophoresis.

**Table 1:** Primers details.

ID	GenBank accession #	Map Location	Locus	Size (bp)	Flurochrome	Forward primer 5’-3’	Reverse primer 5’-3’
**A**	T58329	3-00-0	301CT1	207	AT 532	CTGAACGCGCCATAAATTCT	AGGAGTTCGTCCCAAGACAA
**B**	BM005489	3-23.5	766ATT1	301	FAM (Fluorescein)	TGCAAAGTCGAAGCAACAAG	GAATGCCATTTGCCTTCA
**C**	R47184	3-32.1	69TGA1	214	FAM (Fluorescein)	CACCTCCGCTAGAGAACTGG	CGAATAGGGCAATCCTGAAA
**D**	DV309356	3-43.7	86AC1	257	AT 532	GCGAATCGGTTCCCATAGTA	ACCCATCGAATTTCCATTCA
**E**	AF324863	3-50.0	217CTT1	257	FAM (Fluorescein)	TGGACTTCCCCAGATGCAATGA	CAACACGGAAGCAAAGTTGA
**F**	L12389	3-57.1	201AAT1	336	AT 550	GATCGTTCGACAGCATCTGA	GGAAAGCTCATCGCCTACTG

Primers details of the 6 microsatellites markers used (modified by [50]). We called the microsatellites A to F for simplification (column ID).

### Parental generation

Eggs of *Ae*. *aegypti* from the colony were synchronously hatched under low pressure conditions, and 400 haphazardly chosen larvae were individually reared in the wells of 12-well tissue-culture plates filled with 3 mL of deionized water. The larvae were fed daily with TetraMin™ fish food (hatching day: 0.04 mg/larva, 1 day old: 0.05 mg/larva, 2 days old: 0.1 mg/larva, 3 days old: 0.2 mg/larva, 4: days old 0.4 mg/larva, 5 days old and older: 0.4 mg/larva). Two days after hatching half of the larvae received a control solution of crushed mosquitoes, and the other half received a solution containing 10^4^ spores of *V*. *culicis* (originating from a stock stored at 4°C) and crushed mosquitoes, Both solutions included the daily amount of food. The concentration of spores was determined with a hemocytometer and a phase-contrast microscope. The number of spores was chosen because in previous experiments it led to close to 100% infection success but very little larval mortality. The pupae were individually placed into Falcon tubes and, once adults, provided with a cotton ball soaked with 10% sugar solution. Two days after emergence pairs of males and females were moved to 180-mL plastic cups for mating. All of the males were from the control treatment. Two, nine and 16 days later, females were allowed to take blood meals on GZ’s arms for 10 minutes. The eggs were collected and stored in an incubator at standard lab conditions. The adults were then killed and stored in Eppendorf tubes at -80° C until the molecular analysis. Once mosquitoes were prepared for the extraction of the DNA (see below), we took 8 μl from the extraction tubes and confirmed the presence of *V*. *culicis* with a hemocytometer and a phase-contrast microscope. For further analysis, we haphazardly chose for each block and each treatment ten females that laid at least 40 eggs (over their three blood meals) and that were heterozygous for at least two microsatellites.

### Offspring generation

We bleached the eggs of all families with 1% household bleach (MR4, Methods in Anopheles Research) to eliminate possible spores of the parasite. The larvae of each family were reared in a petri dish containing 100 mL of deionized water and were fed daily with TetraMin™ fish food (hatching day: 0.06 mg/larva, age 1: 0.08 mg/larva, age 2: 0.16 mg/larva, age 3: 0.32 mg/larva, age 4: 0.64 mg/larva, age 5 and older: 0.32 mg/larva). The pupae of each family were moved to a cage containing a 10% sugar solution. Once all the pupae had emerged, we killed the mosquitoes and stored them individually in Eppendorf tubes at a -80° C. An average of 19 offspring per family (4 to 33) were haphazardly chosen for the molecular analysis.

### Molecular analysis

#### DNA extraction

A different procedure was used for the parents and the offspring because of the different sample sizes of the offspring and the parental generations. For the offspring, we extracted total DNA with QIAGEN DNEasy® 96 Blood & Tissue Kit (plate extraction) following the protocol of the manufacturer. The samples were randomized, and each DNA extraction plate contained 95 mosquitoes and one negative DNA extraction control (*Ixodes ricinus* tick). For the parental generation we extracted total DNA using the QIAGEN DNEasy® Blood & Tissue Kit (individual tubes).

#### PCR

PCR amplification of the selected microsatellites was performed in 96-well plates with a thermocycler Mastercycler (Eppendorf). The PCR mix for one reaction contained 5 μL 5X GoTaq® Reaction Buffer (Promega), 0.5 μL PCR Nucleotide Mix (10 mM of each dNTP) (Promega), 1 μL of each primer (10 μM), 0.2 μL GoTaq® G2 DNA Polymerase (Promega) and 2 μL DNA template. A final volume of 25 μL was reached with PCR-grade water. Each plate contained the DNA of 88 samples randomly assigned, 4 intra-plate replicates, 3 inter-plate replicates and 1 negative control.

The thermocycling conditions of the PCR amplification for microsatellites A, B, D and F included a denaturation step at 94°C followed by 30 cycles of 94°C denaturation for 45 s, 60°C annealing for 45 s and 1 min of extension at 72°C, followed by 10 min final extension at 72°C. For microsatellites C and E, the annealing temperature was changed to 55° C and 51° C, respectively. The PCR products were sent to Microsynth AG for the capillary electrophoresis genotyping.

#### Genotyping and recombination fraction

We used the program GeneMapper Software (version 4.1) to obtain the genotypes of the samples. We used the CRI-MAP software version 2.507 (see S1 File, S2 File) to determine the recombination rate in each mother (i.e., the proportion of offspring that carried a combination of alleles that she did not have). 624 offspring were screened (252 for block 1 and 372 for block 2). The recombination rate for each of the 15 pairwise combinations of microsatellites was defined as the ratio of the number of recombinant offspring and the total number of offspring. Recombination between the loci A and F and between A and D of the uninfected treatment could not be detected with CRI-MAP.

### Statistical analysis

We analysed the number of recombinant and non-recombinant offspring per family with a GLME (generalized linear mixed effect model) with binomial distribution. We included in the analysis the experimental block, and the infection status of the mother, the expected recombination rate between pairs of microsatellites and their interaction as fixed factors. We included the mother as a random factor to control for the multiple microsatellite pairs per mother. The expected recombination rate was obtained from a transformation of the genetic distance between pairs of microsatellites given in the literature [51, 52] with Kosambi’s function, which corrects for interference and multiple crossing-overs that can occur at large genetic distances [53–55]. The transformation also takes into account that the probability of recombination between two loci increases with the distance between them [56]. All statistical analyses were performed with R version 3.4.2 and the RStudio interface version 1.1.183. The lme4 [57] and car [58] packages were used for the mixed effect models.

## Results

**Fig 1:**
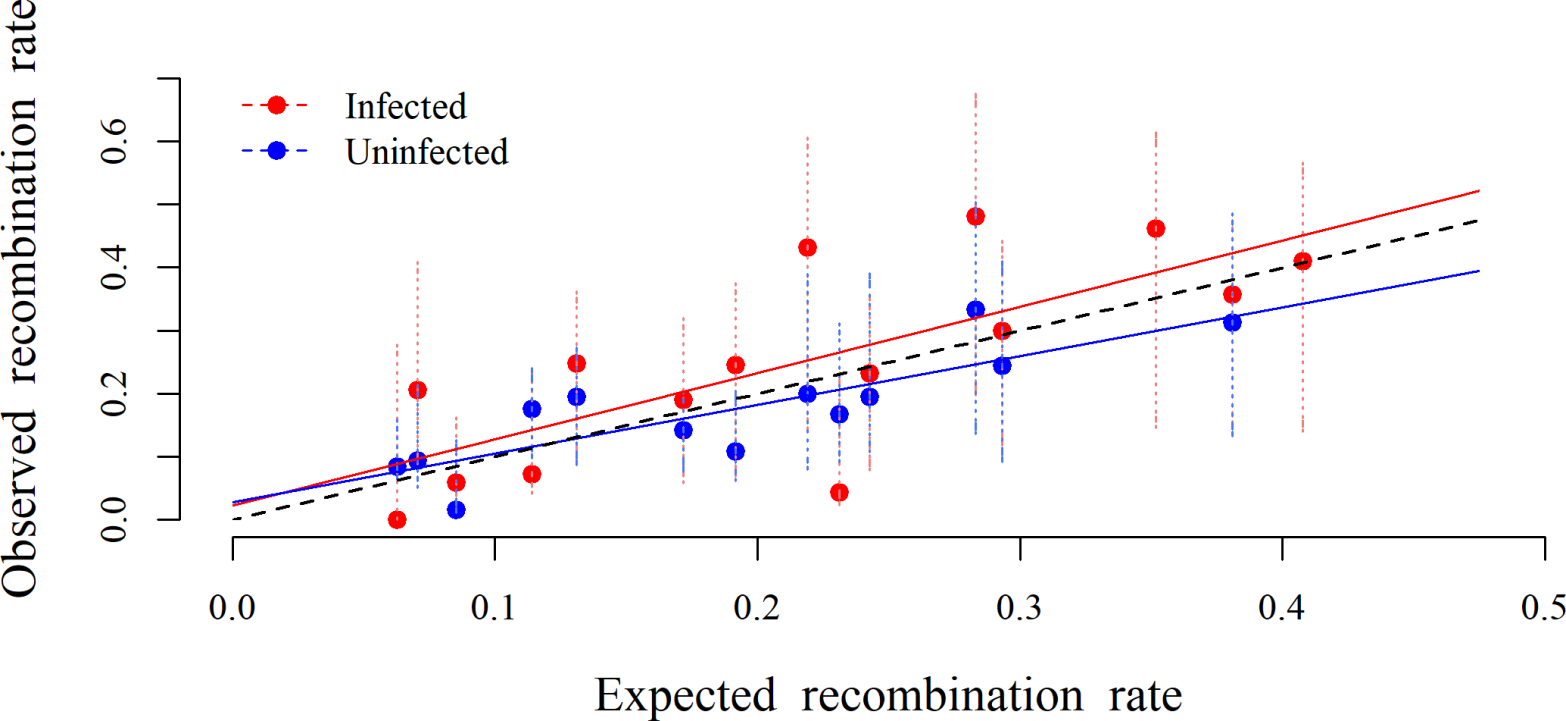
Observed recombination rate of microsporidian-infected and uninfected *Aedes aegypti* as a function of the expected recombination rate. Each red and blue circle (and the corresponding red and blue dashed lines), represents the mean ± 95% confidence interval of the proportion of recombinants in the screened offspring (observed recombination rate) per couple of microsatellites of the respectively infected and uninfected treatment. The red and blue solid lines represent the regression line for the infected (y = 0.02296 + 1.04900x) and uninfected (y = 0.02716 + 0.77370x) individuals. The black dashed line represents the 1:1 reference line (y = x).

The recombination rates between pairs of microsatellites ranged from 0% to 50%. Infected mothers had higher recombination rates than uninfected ones in 10 out of the 13 pairs of microsatellites in which recombination was detected in both treatments (Fig 1). If there were a probability of 0.5 that infected individuals have a higher rate of recombination than uninfected ones, this or a more extreme pattern would occur with a probability of 0.046.

Observed recombination rate increased with expected recombination rate for uninfected and infected individuals (main effect of infection: χ^2^ = 18.49, df = 1, p<0.001), but in the infected individuals the recombination rate increased more steeply with expected recombination than in uninfected individuals (interaction infection*expected recombination: χ^2^ = 3.89, df = 12, p = 0.02). Indeed, between the microsatellite couples A-B and A-C, infection nearly doubled the recombination rate. The experimental block had little impact on recombination rate (χ^2^ = 2.75, df = 12, p = 0.15).

## Discussion

Our data indicate a phenotypically plastic increase of recombination rate of the mosquito *Ae*. *aegypti* in response to infection by the microsporidian *V*. *culicis*.

Our study corroborates previous ones in animals and plants. Females of *Drosophila melanogaster* challenged with a variety of parasites plastically increased the proportion of recombinant offspring they produced [38]. The increase in recombination rate was found with a Gram-positive bacterium, Gram-negative bacterium and a parasitic wasp. A similar response was found in the leaf tissues of *Arabidopsis thaliana* and *Nicotiana tabacum* after infection by *Peronospora parasitica* [35, 37]. These results found with bacterial, fungal and animal parasites suggest that a plastic increase of recombination rate could be a general response to parasitism, although bacterial infection does not appear to increase recombination rate in house mice [39].

In addition to the experiment mentioned earlier, where the red flour beetle increased its recombination rate after several generations of coevolution with a microsporidian parasite [20, 22], several studies support the idea that parasites select for greater recombination. Two examples are that sexual populations of the freshwater snail *Potamopyrgus antipodarum* are less frequently infected than asexual ones [59–61], and that the coevolution between *Caenorhabditis elegans* and *Serratia marcescens* increases the level of outcrossing (and thus, presumably, genetic recombination) [62] and constrains the spread of self-fertilization [63]. Our study adds to previous literature showing that recombination rate can change immediately and plastically rather than increasing evolutionarily over time.

Several potential mechanisms may underpin the increased recombination rate we measured. Stressful conditions (e.g. a parasitic challenge), increase the cross-over events and may have severe effect on the oogenesis of females [64, 65]. Further, parasite infection drastically changes the physiology of the host leading to cascade of events. These include a change in the microbiota, the modification of the immune system and of the level of oxidative stress with the activation/inhibition of molecular pathways [66–69]. Thus, the diversion of resources to face the infection may cause energy burden and higher rate of damages to the DNA, and eventually causing higher recombination rate. In our system, the reduction in the availability of resources of the host is likely to have an important role in the modified recombination rate considering that microsporidia steal ATP from host cells [70, 71].

A possible adaptive explanation for this response is that increased recombination in infected individuals increases the diversity and the frequency of novel genotypes in their offspring. This may help infected individuals to protect their offspring against infection by the same parasite, if the success of the parasite depends on several genes of its host’s genotype. As the parent’s parasites have been able to overcome its genetic resistance, offspring with unfamiliar genetic combinations are expected to be more resistant against the same parasites. This idea underlies a version of the Red Queen Hypothesis, which states that the pressure imposed by parasites contributes to the maintenance of sex and genetic recombination when the offspring are likely to encounter parasite from their mother and not by random chance [17]. Moreover, since the rate of recombination [72–75] and resistance against parasites [76–78] are heritable traits that can respond to selection, strong parasite pressure can select for a higher rate of recombination [79–82].

Recombination rate may therefore evolve in response to parasites’ selection and directly influence fitness-related traits in the host. Despite this possibility have not been formalized, some theoretical results suggest that the evolution of plastic recombination may occur in diploid organisms in the presence of maternal effects on fitness [17].

In contrast to previous work, we focused on microsatellites rather than visible mutant genetic markers [38], which themselves can have fitness costs [83]. This feature of our study allowed us to investigate variation in recombination rate due to parasite infection without searching for associations with specific genes and mechanisms which would have require a more precise scale [23]. There is a general absence of empirical and theoretical work exploring recombination rate plasticity in response to parasites. Experimental tests on *Ae*. *aegypti* with different parasites and the use of new host-parasite systems will be fundamental to expand our knowledge and confirm the findings on this understudied topic.

## Conclusions

In contrast to most studies, ours shows a plastic response of host’s recombination in response to a parasite infection, suggesting an adaptive role of recombination against parasites. We emphasize that recombination rate can be plastic, and we hypothesize that this plastic response may help the host to protect its offspring against its parasites escaping the detrimental parasite pressure without directly involving immune or immune-related response and genes. The effect of parasite infection on the plasticity of recombination and the consequences for genetic diversity will be critical to understand how host and parasite populations co-evolve. Furthermore, since *Ae*. *aegypti* is an important vector of many parasites of humans, theoretical and practical investigations on its plasticity in recombination rate and the potential for ecological and evolutionary feedbacks will be required.

## Supporting information

S1 File CRI-MAP documentation. (PDF)

S2 File CRI-MAP example. (PDF)
