# Supplementary material for: The effect of parasite infection on the recombination rate of the mosquito Aedes aegypti
Source: PLoS One. 2018 Oct 9;13(10):e0203481. doi: 10.1371/journal.pone.0203481 (PMC6177114; doi:10.1371/journal.pone.0203481)
Supplement: S1 File — (PDF) [file pone.0203481.s001.pdf]

Documentation for CRI-MAP, version 2.4 (3/26/90)

Phil Green, Kathy Falls, and Steve Crooks

INTRODUCTION

The main purpose of CRI-MAP is to allow rapid, largely automated construction of multilocus linkage maps (and facilitate the attendant tasks of assessing support relative to alternative locus orders, generating LOD tables, and detecting data errors). Although originally designed to handle codominant loci (e.g. RFLPs) scored on pedigrees "without missing individuals", such as CEPH or nuclear families, it can now (with some caveats described below) be used on general pedigrees, and some disease loci.

This version of CRI-MAP is distributed as source code in the language C, on a 360K DOS formatted diskette. To use it, you will need to obtain access to a C compiler for your computer, and compile the program yourself (see the section **GETTING STARTED**, below). The present version of the program adheres fairly rigidly to the conventions of "standard C" as described in Kernighan and Ritchie (1978) (the main exception being that some variable and function names have more than 8 significant characters), and should be compatible with most C compilers. The program was developed and tested using vers. 2.0 and 2.3 of the VAX C compiler, on a Microvax II with 5 Mb memory under the MicroVMS operating system. It has been successfully ported to a number of other computers, including other VAXes, SUN and Apollo workstations, and the Mac II.
CRI-MAP requires a lot of memory; it is desirable to run it on a computer with at least 1 Mb (RAM or virtual memory) if you will be analyzing more than 10 loci simultaneously. It may be possible to run it on an IBM AT under DOS if your data set is small and you reduce the default memory allocations, although we have not tried this yet.

A small data set with several chromosome 7 markers is provided with the program for the purpose of testing it (only). I would appreciate being informed of any difficulties in implementing the program, bugs, errors or gaps in the documentation, or suggestions for improvement.

Historical note:
Version 1 of CRI-MAP was originally written by me in the summer of 1986 in the language APL; the portion of that version which does maximum likelihood estimation for a fixed locus order was based on algorithms developed in collaboration with Eric Lander (Lander and Green, 1987). Collaborative's chromosome 7 map (Barker *et al.*, 1987) was constructed using that version of CRI-MAP, running on an IBM XT. In the summer of 1987 parts of the original version were translated into C, with the help of Steve Crooks, and used in constructing the genome map published in *Cell* (Eric and his group at MIT independently constructed maps using the program MAPMAKER). At this time I also discovered the "layered EM" maximum likelihood search method, described in (Green, 1988). In January, 1988, I worked out a much faster algorithm for likelihood calculation (the method of switch algebras -- actually a family of related algorithms), also described in (Green, 1988). I have since written and tested the code for these new algorithms and incorporated them into version 2.0 of the program; Kathy Falls has worked on improving various other parts of the program. Versions subsequent to 2.3 were developed by me at Washington University. I soon hope to incorporate a full likelihood analysis for pedigrees with missing individuals, and allow for disease loci with incomplete penetrance. Any user feedback will be gratefully received, and I will do my best to incorporate suggestions into future versions of the program.

Phil Green
Dept. of Genetics
Box 8232
Washington University School of Medicine
St. Louis, MO 63110
(314) 362-5192
FAX: (314) 362-4137
EMAIL: pg@genome.wustl.edu

CONTENTS

Using CRI-MAP with general pedigrees and disease loci

Getting started

File structures
- .gen file
- .par file
- .dat file
- .ord file

Program options
- **all**
- **build**
- **chrompic**
- **fixed**
- **flips*n***
- **instant**
- **merge**
- **prepare**
- **quick**
- **twopoint**

Technical notes

Changes incorporated in versions 2.2 - 2.4

Bibliography

USING CRI-MAP WITH GENERAL PEDIGREES AND DISEASE LOCI

CRI-MAP can only handle disease loci to the extent that genotypes are known: this means that with autosomal dominant loci, affected individuals are to be scored as heterozygotes at the disease locus, while unaffecteds are scored either as homozygotes for the normal allele (if you are willing to assume complete penetrance for that individual), or as genotype missing (alleles both 0) otherwise. For recessive loci, only affecteds and obligate carriers should be scored for the disease locus.

With general pedigrees, CRI-MAP deduces missing genotypes where possible, and computes a likelihood based only on analysis of the known or deduced genotypes. (Partial genotypes, i.e. those in which only one allele is known or deducible, are also utilized in the likelihood calculation to the extent possible).

This likelihood is the usual likelihood (as defined for example by Ott (1985)) when the pedigree data are "complete" (i.e. genotypes are known or can be deduced at every locus, for every individual having descendants in the pedigree).

If data are missing for an individual at a particular locus, and the possible genotypes at that locus (i.e. those genotypes compatible with the genotypes of ancestors and descendants) include a homozygous genotype, then all meioses in that individual are treated by CRI-MAP as uninformative for that locus. A full likelihood analysis (as provided for example by LIPED or LINKAGE) would instead consider in turn each possible genotype at the locus, assign relative probabilities to each based on the genotypes of ancestors and descendants, and compute a likelihood which is the weighted sum of the likelihood expressions for each particular choice of genotype. CRI-MAP thus ignores some of the available information. In cases that we have examined, however, the information loss appears to be small.

If the missing locus genotype is in an "original parent" (i.e. an individual with no ancestors in the pedigree), then in a full likelihood analysis the population allele frequencies are used to assign probabilities to various possible genotypes. These enter into the likelihood by influencing the probability that the allele in any child of the original parent is derived from that parent. CRI-MAP does not make use of allele frequencies; for any allele in a child of an original parent, CRI-MAP determines the parental origin when this can be deduced from the other genotype information, but otherwise assigns equal probability to the two possible parental origins for the allele. In fact, little information is lost by this procedure, except when the allele is rare. For a rare dominant disease, if one parent is known to be affected, the other should therefore be scored as unaffected (rather than as missing, for example).

It should be noted in any case that the frequencies of RFLP alleles have usually been estimated in a different population (e.g. the CEPH family parents) from the population from which the disease family is drawn. It is our experience that allele frequencies may vary dramatically between populations.

Therefore it may be inappropriate to perform a full likelihood analysis of disease linkage, if the results of that study depend in an essential way on parameters estimated from another population. A somewhat more limited analysis which makes no assumptions concerning allele frequencies, such as that given by CRI-MAP, may be preferable. If (for reasons of power) a full likelihood analysis is necessary, that analysis should be performed using a range of allele frequencies at each locus, in order to ensure that the results do not depend in an essential way on these frequencies.

Finally, it should be noted that whenever some available information is arbitrarily excluded, as is the case with CRI-MAP's analysis of general pedigrees, there is a potential that artifactual biases in parameter estimates can be created. In a preliminary examination of this possibility (cf. Goldgar *et al.*, 1989), we have compared the results of CRI-MAP with those of a full likelihood analysis program (LINKAGE) for all pairs of a set of 20 linked loci, in a data set having 136 pedigrees, most with missing individuals. The information loss (as estimated by comparing the effective number of informative meioses for the two programs) averaged about 15%, and the estimated recombination fractions were almost exactly the same. This suggests that bias is not significant and information loss is small, at least in this particular data set.

However, the possibility of bias clearly needs to be considered more thoroughly by means of simulation studies involving multiple loci, and we are in the process of doing this. I would recommend that you check the extent of information loss and/or possible bias in your data set by comparing the results of CRI-MAP to those of a full likelihood program for small sets of loci.

GETTING STARTED

1. Put all files on the diskette provided into a single directory on your computer.

2. Compile the source code files (i.e. those whose names end in .c), and link them together with your C stdio and math libraries, producing an executable file called crimap. There are slight differences in the conventions used by some C compilers which may make it necessary for you to modify some of the source files before they will compile correctly; usually these will be evident from the error messages generated by the compiler. For example, if your operating system is case-sensitive (e.g. UNIX), you will need to rename all files in lower case (DOS converts all file names to upper case). We have declared some stdio and math library functions in the crimap functions where they are used; if your compiler objects to this, you will need to delete the offending declarations.
Note to VAX C users: there is a bug in the optimizer of the VAX C compiler, ver. 2.3 (and possibly in other versions), which causes it to compile the source file "e_conv.c" incorrectly. When the program is run, this will result in an incorrectly created phase-known data structure in the .dat file (see below). To avoid this, compile e_conv.c with the /NOOPTIMIZE option. All other source files should be compiled with the default /OPTIMIZE option.
Also see comments on "underflow" under **TECHNICAL NOTES**.

3. The command format for running the program is
crimap {chromosome number} {option}
Example:
crimap 7a twopoint
The option name must be entered in lower case letters. Chromosome "number" (which may consist of any string of digits, possibly followed by letters -- for example 7p, or 17nf, or 0a) may be replaced by the name of the parameter file (described below); for example,
crimap chr7a.par twopoint
You must provide a .gen file (named in accordance with the conventions described below, and residing in the same directory as CRI-MAP) which contains the raw genotype data, *and* run the **prepare** option first in order to create the other files required by the program.
All program output (apart from specific information written to one of the four files described in the next section) is displayed using the "printf" function in C. It will thus be displayed on the terminal (unless redirected to a file by means of commands to the operating system) if the program is run interactively, or written to a log file if the program is run in batch mode. (The latter procedure is the most convenient way to make a copy of the output. In UNIX and some other operating systems, one can simply redirect the output to a file.) The **prepare** and **merge** options are the only ones requiring interactive input.

4. As a test run with the chromosome 7 data set, chr7a.gen, provided with the program, use the command
crimap 7a prepare
to create a .dat file and a .par file for subsequent use by the option **all**, with the loci 2 8 9 10. (Specify any two of these as the "ordered loci", and the other two as the "inserted loci".)
Use default values for the other parameters.
(**NOTE:** If the program stops prematurely, displaying the message "Your compiler uses a different size for integers; see documentation for changes that will have to be made in the source code", then you will need to make certain minor modifications in the source files and recompile. See "16 bit integers" under **TECHNICAL NOTES**, below.
If the program stops prematurely, displaying the message "ERROR: ALLOCATION FAILED IN MORECORE", then the program's default memory allocations exceed what your operating system can provide. Reduce the value of nb_our_alloc in the .par file (using **prepare**, or a text editor) and see "Memory management" under **TECHNICAL NOTES**, below.
Then run CRI-MAP with the command line

crimap 7a all

The program prints out parameter values, tests all 12 possible orders of these 4 loci, and finally prints out a sorted list of all orders whose $\log_{10}$likelihood is at least that of the best order, less 3.0.
You should get the answer:

2 10 9 8 -97.937
2 10 8 9 -100.839

The run to evaluate these 12 orders should take less than a minute on most workstations or moderately powerful personal computers (for example, about 50 secs on a Mac II, about 25 secs on a MICROVAX II without a numerical coprocessor, about 8 seconds on a SUN 3/60 or 2 seconds on a 4/60; exact times will depend on the efficiency of the C compiler used as well as the computer).

FILE STRUCTURES

CRI-MAP uses four different types of files. These all have names of the form chr*x.y*, where *x* is the chromosome "number" and the suffix *.y* is one of

.gen	the raw genotype data file
.par	the parameter file
.dat	the processed data file
.ord	the orders file, or "orders database"

The user must supply the .gen file. The .dat and .par files are created using the option **prepare**, and must be constructed before using any other CRI-MAP options (except **merge**). The .ord file is initialized by **prepare**, but required only for the map building options (**build**, **instant**, and **quick**). You will need to learn about the structures of the .gen and .par files, but can ignore the descriptions of the other file types if you wish.
Each file is in ASCII format, and can be edited with a text editor. For readability, the user can insert additional blank or end of line characters into these files if desired, since the C statements which read the files treat any string of such characters as a single "white space" delimiter between data items. All data items are either numbers or character strings containing no blank spaces; they must be separated by white space delimiters.
In the following descriptions, brackets {} are used to enclose data items appearing in the file but do not themselves appear in the file.

.gen file

The data items in this file are as follows:
{# of families}
{# of loci}
{locus name1} {locus name2} . . . (each name consisting of at most 15 characters)
For each family:
{family ID} (consisting of an arbitrary character string)
{# of members}
For each member in the family:
{ID #} {mother's ID #} {father's ID #} {sex: 0 if female, 1 if male, 3 if unknown}
{locus1 allele1}{locus1 allele2}{locus2 allele1}{locus2 allele2} . . . .

The pedigree structure must be completely specified; this means that for any individual with an ancestor in the pedigree, both parents must be assigned ID #'s and must appear in the file. The family ID may be any string of characters (without embedded blanks) but individual IDs must be numbers. For "original parents" (individuals without ancestors in the pedigree), the mother and father IDs are coded as 0. Alleles must be represented by integers, with missing alleles scored as 0. To handle new mutations in a dominant disease gene correctly, ancestors of the mutant individual should have their disease locus genotypes coded as missing. To handle non-pseudoautosomal X linked loci, a dummy allele number (e.g. 9) must be created and assigned as the second allele for all males in the pedigree.

Example: For a data set with two loci, LOCA and LOCB, scored on the single pedigree depicted on the next page, the corresponding .gen file would be
1
2
LOCA LOCB
P100
6
1001 0 0 0 1 2 2 2
1002 1001 1009 0 1 2 1 2
1003 0 0 1 1 1 1 2
1004 1002 1003 0 1 2 1 1
1005 1002 1003 0 1 1 2 2
1009 0 0 1 0 0 0 0
Note that the missing maternal grandparent has been assigned an ID of 1009 and included in the file.

.par file
This file may either be created using the **prepare** option, or directly using a text editor. The format has been changed effective with version 2.4, and differs somewhat from that of the other files.
Each line contains a parameter or variable name and its value(s) (separated by spaces), and concludes with an asterisk *. The parameters may appear in any order within the file. If a parameter is omitted (either by omitting the corresponding line altogether, or by omitting the value between the parameter name and the asterisk), then CRI-MAP will automatically assign a default value to the parameter.
In the following descriptions, each line ending in an asterisk is displayed as it would appear in the .par file, and gives a parameter name together with its default value(s) (if any). Any number of hap_sys, hap_sys0, and fixed_dist lines may appear in the file. The other parameters should appear only once. (If they appear more than once, only the last specified value will be used).

dat_file chr*x*.dat *
gen_file chr*x*.gen *
ord_file chr*x*.ord *

These give the names of the .dat, .gen, and .ord files to be used in the analysis. When the name of the .par file is *not* of the form chr*x*.par, the default names for the above files are obtained by replacing the ".par" (which must always appear) in the name of the .par file with ".dat", ".gen", or ".ord", respectively. (This is sometimes useful if one wants to keep all files in a separate directory from the program itself, in which case the name of the .par file passed to crimap may include the full path name; the default names for the other files will then include the same path).

nb_our_alloc 3000000 * (Note: OK for 50 markers)

The initial memory allocation (in bytes). The default value will suffice for most runs, except with a very large number of loci, and in fact is much more than is usually needed for analyzing a small number of loci. If additional memory is needed during the run, it will be allocated automatically (up to the limits of your system); however it is advantageous to choose values for the initial allocation which will suffice for the entire run. See "Memory management" under **TECHNICAL NOTES**.

SEX_EQ 1 *

SEX_EQ is 1 if recombination rates are assumed equal in the two sexes, 0 if sex-specific rates are to be allowed in each interval.

TOL .01 *

The tolerance for determining convergence of the layered EM algorithm; when $\log_{10}$likelihoods from successive "phase unknown" iterations increase by less than this amount, iteration terminates. The tolerance used to detect convergence in the "non-informative locus" part of layered EM, and in the option **twopoint** is TOL/10. In extensive tests using our RFLP CEPH family data sets (several hundred maximum likelihood estimations) the default value .01 was always found to be adequate (it was occasionally not adequate when

ordinary EM was used as the search method, instead of layered EM). If you are concerned about the possibility that this may not be stringent enough for your data set (for example, if the likelihood surface is relatively flat), try using .001 instead; the linear nature of EM convergence guarantees that, if the estimates had not converged with TOL = .01, then a substantial improvement in likelihood should be apparent with the more stringent tolerance. If such an improvement is seen, use successively smaller values for TOL until no further improvement in the likelihood results.

PUK_NUM_ORDS_TOL 6 * (Note: is actually PUK_NUM_ORDERS_TOL)
Applies only to the option **build**; gives the maximum number of orders allowed in the current map, in the phase unknown part of the analysis.

PK_NUM_ORDS_TOL 8 * (Note: is actually PK_NUM_ORDERS_TOL)
Similar to PUK_NUM_ORDS_TOL, but applies instead to the phase known analysis in **build**. If 0, the phase known analysis will be skipped entirely during map building.

PUK_LIKE_TOL 3.0 *
The tolerance for discarding locus orders. If the $\log_{10}$likelihood of an order is less than the $\log_{10}$likelihood of some other order for the same loci by an amount exceeding PUK_LIKE_TOL, that order is discarded (or not printed). Used by map building options, **all**, and **flips*n***. With **twopoint**, LOD tables are displayed only for locus pairs whose LOD exceeds PUK_LIKE_TOL.

PK_LIKE_TOL 3.0 *
As above, but applies only to analysis of the phase known data in the option **build** (and is not used by the other options).

use_ord_file 0 *
This parameter applies only to the options **all** and **flips*n***. When it is 1, the orders generated by those options are prescreened against the .ord file to eliminate orders incompatible with the orders database, prior to computing likelihoods. When use_ord_file is 0, the information in the orders database is not used.

write_ord_file 1 *
Applies only to the option **build**. When it is 1, the results of the current **build** run are used to update the orders database. When it is 0, the orders database will not be updated (but will still be used to prescreen orders during the course of the run).
ordered_loci {index # of 1st ordered locus} {index of 2d ordered locus} . . . *
inserted_loci {index # of 1st inserted locus} {index of 2d inserted locus} . . . *
Note: locus indices start at 0.

For all options except **twopoint**, the "ordered_loci" are assumed to be in their known, unique order; the remaining loci, called the "inserted_loci", are to be placed in the framework defined by the ordered loci. For the options **chrompic**, **fixed**, and **flips*n***, there are no inserted loci. For **twopoint**, if both ordered_loci and inserted_loci are specified, then LOD tables are only computed for pairs of loci for which one is in the ordered list and the other is in the inserted list. Otherwise the analysis uses all pairs of loci in the specified list (ordered or inserted).

hap_sys {index # of 1st locus in system} {index # of 2d locus} . . . *
hap_sys0 {index # of 1st locus in system} {index # of 2d locus} . . . *
Each haplotyped system is a list of loci (for example, different RFLPs detected by the same probe) which are to be grouped together in an analysis. The first locus in any system is called "primary", and the remaining loci are called "secondary". When the parameter use_haps (see below) is 1, the secondary loci in a system "tag along" with the primary locus whenever ordered sets of loci are constructed. The operations which construct new orders (for example, by inserting a new locus into the map, or by permuting a collection of loci) utilize only the

primary loci; once the order is constructed, however, it is "filled out" by inserting secondary loci immediately following the corresponding primary locus, prior to calculating likelihoods and map distances. Secondary loci are automatically deleted from the input lists of ordered loci and inserted loci. Thus any system which is to be included in the analysis must be represented by (at least) its primary locus.
For systems specified using hap_sys, the loci within a system are treated as independent in all calculations; i.e. they are not forced to have 0 recombination fraction. In particular, intralocus recombinants between loci in the same system are permitted. For systems specified using hap_sys0, distances within the system are forced to 0 (the program will stop, displaying the message ERROR: 0 likelihood, if there are in fact intralocus recombinants). Example: two haplotyped systems, the first having loci 3, 4, and 5 with distances not forced to 0.0, and the second having loci 9 and 11 with distances forced to 0.0, would be entered in the .par file as

```
hap_sys  3 4 5 *
hap_sys0         9 11 *
```

use_haps   1 *

When use_haps is set to 1, haplotyping is performed; when it is 0, any haplotyped systems specified in the .par file are ignored (i.e. the input lists of loci are taken as is, and no secondary loci are deleted or inserted).

fixed_dist {rec. frac.} {index # of 1st locus} {index # of 2d locus} {sex (optional)} *

For the options **fixed** and **chrompic** (only), the recombination fraction between a pair of adjacent loci may be held fixed using fixed_dist. If the recombination fraction to be fixed is sex-specific, specify the sex as 0 for female, or 1 for male. If either locus is part of a haplotyped system, it must be the primary locus from its system (it is not possible to force a distance within a haplotyped system, except by using hap_sys0 as described above). Note: any recombination fractions held fixed, either using fixed_dist or hap_sys0, are flagged by an asterisk in the map displayed following the analysis.

The last line of the .par file must contain the single word **END**.

Example: if you wished to construct a .par file chr7a.par to perform the **all** analysis described in **GETTING STARTED**, above, the following three lines would suffice (since default values are to be used for all other parameters):

```
ordered_loci   2 8 *
inserted_loci   9 10 *
END
```

.dat file

This file, which is created by the program (using the **prepare** option), not the user, has two data structures, each having the following data items:
{# of loci}
{# of families}
{family id1}
{family id2}
. . . (may be any character strings not containing blanks)
{locus name1} {locus name2} . . . (each locus name consists of at most 15 characters, with no embedded blanks) For each family:
{# of chromosomes}
{phase chromosomes: each is a character string of length numloci with values 0, 1, or X, denoting the phase}
{# of switches}
For each switch:
{index of locus affected by the switch (in this file, locus indices start at 1, not 0)} {string of length numchroms, with entry 1 if the corresponding chromosome is affected by the switch, 0 otherwise}

The first such data structure has the "phase known" data: there is 1 "family" with all the chromosomes in the data set, and 0 switches. All loci of unknown phase are given phase X, as are loci on the chromosomes of children of identical heterozygotes (the latter restriction is necessary to avoid bias in the estimation of recombination fractions, cf. Ott (1985)).
The second data structure contains the full phase information for the data set, arranged by families. (For definitions of switches and phase chromosomes, see (Green, 1988)).
The "phase known" data structure is used only by the option **build**.

.ord file
(For definition of terminology, see description of the **build** option, below.)
{# of orders objects}
For each orders object:
{# of orders} {# of loci}
For each order in the orders object:
{index of 1st locus in the order} {index of 2d locus in the order} ...

PROGRAM OPTIONS

all
Finds $\log_{10}$likelihoods for all locus orders which result from placing the inserted loci in all possible positions with respect to the ordered loci (see description of the .par file, above, for definition of these terms). The program finds the order with the highest $\log_{10}$likelihood L and prints out, in decreasing order of $\log_{10}$likelihood, those orders whose associated $\log_{10}$likelihoods are greater than or equal to L - PUK_LIKE_TOL. In particular, when the number of ordered loci is two, all possible orders of the specified loci are analyzed. When using this option, keep in mind that the number of orders analyzed is n!/f!, where n is the total number of loci and f is the number of ordered loci (and ! denotes factorial), and that analysis of all 360 orders of a typical set of 6 loci on chromosome 7 takes about 30 mins on a MICROVAX II; thus it is probably impractical to use this option with more than 6 inserted loci, except on a much faster computer. When use_ord_file is set to 1 in the .par file, any orders incompatible with the orders database are eliminated prior to calculating likelihoods, which may make it feasible to use **all** with larger sets of loci.

One convenient use of this option is to position a new locus (which would be specified as the single inserted locus) with respect to a set of loci of known order.

build
Builds a map by sequential incorporation of loci. This is the main option used for RFLP map construction.

To describe the method used in greater detail, define an "orders object" to be a collection of loci, together with a set of permissible orders for those loci. The "orders database" contains a collection of orders objects.
At any given stage during the map construction "the map" (designated "current_orders" in the program source code, and output) consists of one such orders object. At the beginning of the run the orders database is empty, and the map consists of the "ordered loci" specified in the .par file. (In the absence of any prior information concerning locus order, such as physical localization data, one usually chooses the "ordered loci" to be a pair of linked and highly informative loci, in order to accelerate the map construction process). The first locus in the list of inserted loci (specified in the .par file) is placed in each possible interval in the map. The resulting locus orders are then tested for compatibility with the database; each order not excluded is subjected to a full maximum likelihood estimation. The order having the highest $\log_{10}$likelihood is found, and any order whose $\log_{10}$likelihood is less than this one by more than a specified tolerance (PUK_LIKE_TOL or PK_LIKE_TOL) is eliminated. The resulting collection of non-excluded orders form an orders object (called orders_temp in the program output). The database is then updated by (1) appending orders_temp to it; and (2) for each orders

object already in the database, eliminating any order which is incompatible with orders_temp. If orders_temp has fewer, or the same number, of orders as does the map, then it becomes the new map and the added locus is deleted from the list of inserted loci. Otherwise the next locus in the list is tried in the same manner. If no locus in the list meets this criterion, the one with the smallest orders_temp is added to the map. Each time a locus is added to the map, the program returns to the beginning of the (revised) list of inserted loci. The orders database is kept in memory during the program run; a copy of it is written to the .ord file every time a new locus is added to the map, so that the program can be stopped and restarted later without loss of the information in the database.

Build first analyzes the "phase known" data (see description of .dat file structure), for which maximum likelihood computation is much more rapid than for the full data set. When the map gets too large (i.e. the number of orders in the map exceeds PK_NUM_ORDS_TOL), **build** proceeds to find a set of uniquely ordered loci, starting with the original ordered loci and adding additional loci which now have a unique placement (during this latter step, the program only makes use of information in the data base; it performs no likelihood calculations). Using this uniquely ordered set of loci as the new "map", the program then proceeds to analyze the full data set, again adding loci sequentially according to the procedure described above. This portion of the program stops when the number of orders in the map exceeds PUK_NUM_ORDS_TOL.
Build then again extracts a set of uniquely ordered loci on the basis of information in the orders database, and prints out sex-specific and sex-averaged recombination fractions and the corresponding Kosambi centiMorgans for these loci. For each remaining locus, it prints out the possible placements with respect to the uniquely ordered loci, along with the $\log_{10}$likelihoods for each placement.

If one starts the map with a pair of unlinked loci, then construction of the map is slower, because in the initial stages the map will consist of two unlinked linkage groups which can be in either orientation with respect to each other so that there are more orders to keep track of (and to place remaining loci in). Conversely, if the initial loci are at 0 recombination fraction (for example, two RFLPs detected by the same probe), then in all subsequent maps the loci will appear in both possible orientations, thereby doubling the number of orders. What is worse, the parts of the program which extract the maximum set of uniquely ordered loci will never get past these two (since no other locus is uniquely placed with respect to them). To avoid this, it is advisable to put these loci later in the list, or (better) to haplotype them using hap_sys or hap_sys0 in the .par file.

It is a good idea to exclude relatively uninformative loci from the initial map construction process: they tend to have multiple possible placements, resulting in a large number of orders to test. Their positions can be determined later using **all**, or **instant**.

With large numbers of loci, it may be necessary to perform several map runs using **build** until one arrives at "the best" set of uniquely ordered loci.
Information in the orders database is cumulative between runs, provided it is not reinitialized using **prepare**; it is not necessary to reinitialize the orders file when using different sets of loci from the same file.

NOTE
The goal of multilocus linkage analysis is to find the locus order having the highest likelihood, and identify alternative orders with comparable likelihoods. Because it is impossible to consider all possible orders for a large set of loci, it is necessary to adopt a strategy which makes decisions on the basis of subsets of the loci. Multiple statistical tests are performed, each with a non-zero probability for Type I error. Because incorrect orders will sometimes have significantly higher likelihoods than the correct one, and the chance of this happening increases with the number of sets of loci which are examined during the map construction process, it is possible that the maximum likelihood order would in fact be rejected by **build** (or any other published method for map construction, for that matter) because for some *subset* of the loci, an alternative order has a significantly higher likelihood. In our experience this happens only very rarely (probably because the tests are not independent), and when it does is usually due to errors in the data itself. Nevertheless, three precautions should be taken to guard against the possibility of an erroneous order being adopted by **build**. First, we usually

construct initial maps using a fairly stringent tolerance for rejecting orders -- PUK_LIKE_TOL at least 3.0 -- and then lower it to 2.0 (and omit the phase known part of the analysis) for construction of the final map. Second, it is advisable to check the final order by running **flips*n*** to look at permutations of successive triples or quadruples of loci. Finally, it is a good idea to construct the map several different times, using different starting pairs of loci and a different sequence for adding the loci each time.

chrompic

Displays the grandparental origins of alleles in each child's chromosomes, for the phase choice having the highest likelihood; gives the relative probability for this phase choice and for the next most likely alternative; gives the number and location of recombinations on each chromosome, and provides, for each chromosome interval defined by the loci, a list of the chromosomes having a recombination in that interval. Flags candidate data errors.

The program begins by finding maximum likelihood estimates of the recombination fractions (for the specified locus order); these are then used to find, for each pedigree in the data set, the particular phase choice having the highest likelihood for that pedigree. (The algorithm used for this purpose is described in (Green, 1988)). For each individual with parents in the pedigree, the chromosomes are displayed (maternal first, then paternal). (The individual's ID # appears to the left of the maternal chromosome.) A '0' (resp. '1') means the allele is of known phase and is of grandmaternal (resp. grandpaternal) origin. For alleles of unknown phase, 'o' and 'i' are used instead, denoting the grandparental origin in the most likely phase choice. '-' means that the locus is noninformative.

To the right of the chromosome appears the number of recombinations.
Beneath the chromosome appear the names of any informative loci which are out of phase with the nearest informative loci on either side; when the loci are closely spaced, these are candidate data errors.

Following the chromosome pictures for all the families is a list of recombinant chromosomes. For each pair of loci, the chromosomes are listed in which there is a recombination between those two loci (and no intervening locus is informative). The recombinant chromosomes are designated by family number, individual ID #, and M or P (for maternal or paternal).

Following the list of recombinant chromosomes is a list of groups of loci not separated by any crossovers in the data set; and finally, the maximum likelihood recombination fractions which were used in computing probabilities of the phase choices.

fixed

For a set of loci in a specified order, finds the associated maximum likelihood recombination fractions and map distances and the corresponding $\log_{10}$likelihood. The recombination fraction between any pair of adjacent loci may be held fixed by using fixed_dist in the .par file.

flips*n*

For each locus order obtained by permuting an ***n***-tuple (***n*** >= 2) of adjacent loci within an initial (reference) locus order, displays the relative $\log_{10}$likelihood (i.e. the $\log_{10}$likelihood of the reference order, minus that of the permuted order). For example, **flips3** will find relative $\log_{10}$likelihoods for all permutations involving triples of adjacent loci. (If ***n*** is omitted it is assumed to be 2). When ***n*** is 2, relative $\log_{10}$likelihoods for all permutations are displayed; for higher values of ***n***, only the permutations whose $\log_{10}$likelihoods are at least that of the original order, less PUK_LIKE_TOL, are displayed.

Note: the total # of orders which must be evaluated is

(m - n + 1)(n! - (n-1)!) + (n - 1)!
where m is the total # of loci; thus it is impractical to use this option with large values of n. However, when use_ord_file is set to 1 in the .par file, permutations incompatible with the orders database are eliminated before calculating likelihoods, which will sometimes make it feasible to use larger n's.

instant
Finds a uniquely ordered set of loci, and the possible placements of the remaining loci with respect to them, using only the information already existing in the .ord file (the .ord file must have been created in a previous **build** run). Log_{10}likelihoods for the different placements of the non-uniquely ordered loci are computed.

merge
Merges two .gen files, having overlapping sets of families and/or loci. The option is run interactively: in response to prompts, the user enters the names of the two files to be merged, and the name for the merged output file. File names must be complete (i.e. the .gen suffix needs to be included). The program compares family IDs and locus names in the two input files, and for families having the same ID compares individual ID #s; it then merges the data from the two files into a single .gen file, taking overlaps into account. A family appearing in both files need not have exactly the same individuals (in which case the output file will have a merged family with all individuals appearing in either input file); the program checks individuals which do occur in both input files to make sure their mother and father ID #s and sex agree, and if they don't displays an error message. If the two files have some loci in common, the genotypes at those loci in the output file for an individual appearing in both input files will be those from the second file.
Note: although the command format for using this option is the same as for the other options, the chromosome number is ignored (although it must appear), and no .par file is needed.
N.B. Be careful when merging files containing, say, CEPH and disease families that there are no spurious matches between family IDs. For this purpose it is useful to use the fact that family IDs may be arbitrary character strings; so for example CEPH family IDs may be prefixed with "CEPH".

prepare
This option (which is run interactively) must be run before any of the other options, to create the .dat and .ord files used by them. It will also create a .par file (or modify an existing one), however once you are familiar with the format of the .par file you will probably find it quicker to create and modify that file with a text editor, rather than use **prepare** repeatedly.

Example of usage:
crimap 7a prepare

Prepare first searches for the .gen and .dat files of the correct name (in the above example, chr7a.gen and chr7a.dat). If there is no .dat file, one is created from the .gen file; if both exist, the program asks whether a new .dat file should be created from the .gen file (select this option if the .gen file is more recent). If neither exists, a message to that effect is given and the program terminates. As the .dat file is being created, family IDs are displayed and any non-inheritances are noted. (These should be checked carefully: a missing data item in the .gen file will generally result in multiple non-inheritances and incorrect family IDs for the subsequent families. If non-inheritances are found, these should be corrected in the .gen file, and **prepare** run again).

Prepare then displays values for several parameter values, including whether to compute sex-averaged or sex-specific likelihoods, and tolerances which control convergence of the maximum likelihood search and the map building process (see description of .par file, above). The displayed values are the defaults (given in the

description of the .par file) or the values specified in any preexisting .par file of the same name. The user is then prompted to change any of these values, if desired.

Any haplotyped systems, or fixed distances (see description of .par file) in a preexisting .par file are then displayed, and you are prompted to enter any new ones if desired. (**prepare** cannot be used to modify or delete systems in the preexisting file; you must use a text editor for this).

Prepare then displays the names of the other CRI-MAP options, and asks which one it should set up the .par file for. Depending on the option chosen, the user is now prompted to specify the indices of the "ordered loci", and of the "inserted loci". The ordered loci are generally fixed in that order during subsequent analysis, while the inserted loci are tried in all possible positions. When nothing is to be assumed about locus order only two ordered loci should be specified. (**twopoint** is a special case; see its description below).

For the map-building options (**build**, **instant**, and **quick**), the ordered loci form the initial map. As the default (in this case only), if requested, **prepare** will sort the loci by informativeness of the phase known data and designate the two most informative loci as the ordered loci, and the remaining loci as inserted. For the options **fixed**, **flips*n***, and **chrompic**, all loci are specified as ordered, and none are inserted. See the description of the .par file, or the relevant option, for further details.

If the option **build** has been specified, **prepare** initializes an orders file, unless one already exists, in which case it asks whether the existing one should be used. Finally, **prepare** asks whether to create a new .par file containing the information you have input. You have the option at this point of aborting the creation of a new file, in which case any preexisting .par file will remain intact.

quick

Similar to **instant**, except that $\log_{10}$likelihoods are *not* computed.

twopoint

Performs twopoint linkage analysis for each pair of loci. In the case of sex specific analyses, the output for each pair appears on three lines: the first has the locus names, followed by the maximum likelihood estimates, f and m, of the female and male recombination fractions, and the sex-specific LODS (= $\log_{10}$like(f,m) - $\log_{10}$like(.5,.5)). The second line has the values of $\log_{10}$like(r,m) - $\log_{10}$like(.5,.5) for r = .001, .01, .05, and all multiples of .05 up through .5.
The last line similarly has the values of $\log_{10}$like(f,r) - $\log_{10}$like(.5,.5).
Only pairs for which the LODS exceed PUK_LIKE_TOL are printed out. (You can get tables for all pairs by setting PUK_LIKE_TOL = 0.0).

When SEX_EQ is 1, the sex-averaged recombination fractions, LODS, and $\log_{10}$likelihoods are given instead.

Haplotyped systems may be used for either locus in a pair. If the loci within a system are not forced to have 0 distance, then maximum likelihood intralocus distances are found simultaneously with the maximum likelihood interlocus distances, and are subsequently held fixed at these values when likelihoods are calculated at the above specified interlocus distances.

If both ordered_loci and inserted_loci are specifed in the .par file, tables are only computed for pairs consisting of one locus from each list. This is convenient, for example, when one has just added data for a new set of loci to a file and wishes to look for linkages with the old loci. If only one list (either ordered_loci or inserted_loci) is non-empty, LOD tables are computed for all pairs of loci within that list.

TECHNICAL NOTES

Memory management
Memory is allocated dynamically by the program, as needed; however, since repeated requests of memory from the operating system (via the C stdio library function malloc) are quite time-consuming, we have adopted a strategy in which two large initial blocks of memory are first allocated. One of these is reserved for the objects comprising the orders database and is apportioned out as needed by the function our_orders_alloc, while the other is used for all other memory requirements and is apportioned by our_alloc. If the amounts initially allocated are insufficient, additional memory is requested from the operating system (via the library function malloc). (When this happens, a statement to that effect is printed). The default initial request of 3 Mb (for our_alloc) will suffice for nearly all runs. If the program requests more memory than the operating system can provide, the program terminates with the message "ERROR: ALLOCATION FAILED IN MORECORE". If this happens, try setting the amount of memory specified in the .par file (using the parameter nb_our_alloc) to the maximum amount the operating system will allow you (and see if your system manager can increase that amount by adjusting operating system parameters). If that fails, you may need to reduce the number of loci being analyzed (often, deletion of a single locus having a large number of switches may suffice), or to split a large pedigree into subpedigrees. I would be interested to learn of cases where that is necessary, since it may be possible to improve the switch algebra algorithm to avoid it.

Underflow
When mapping large numbers of loci, the individual family likelihoods involve products of many factors numerically less than 1, and so can become quite small. If possible, at compilation time you should select an option for representation of floating point numbers which allows them to be as small as possible. With VAX C, this is done by using the /g_float option with the compile command cc, and then linking with the vaxcrtlg library.

16 bit integers
C compilers usually represent integer variables with either 16 or 32 bits. The source code provided to you assumes that you have a "32 bit" compiler. If, when you first try to run the program, it terminates prematurely, displaying the message "Your compiler uses a different size for integers; see documentation for changes that will have to be made in the source code", then you have a 16 bit compiler. To get the program to run correctly you must then make the following two changes in the source code and recompile:
(1) in the file "defs.h", replace the line

```
typedef int INT;
```

by

```
typedef long INT;
```

(2) Consult the manual for your compiler and determine the name of a library memory allocation function which takes long integers, instead of integers, as arguments (the function malloc takes integer arguments). The name of this function will be something like "mlalloc" (Lightspeed C) or "_halloc" (Microsoft C, ver 4.0 or later). Whatever it is, substitute it for "malloc" throughout the source files our_allo.c and our_orde.c. If your compiler does not have such a function, you will be unable to run CRI-MAP.

CHANGES IN VERSION 2.2 - 2.4
The main change in version 2.2 is the incorporation of a more efficient "switch algebra" algorithm which in many cases (particularly for large pedigrees) substantially reduces memory requirements and running time. Thus for many runs, even with a relatively large number of loci, the default memory request of 3 MB (for nb_our_alloc in the .par file) is overly generous.

Version 2.3 incorporates a better switch algebra algorithm which further reduces memory and running time. Additional changes have been made which should make this version compatible with most C compilers.

Version 2.4 incorporates the following changes:

(1) Haplotyping: it is now possible to constrain the loci in a haplotyped system to be at 0 distance from each other for all likelihood calculations. In addition, haplotyping can be used with all of the options; in particular, **twopoint** LOD tables can be generated for pairs of haplotyped systems. Haplotyped systems are now specified in the .par file (and there is no longer a .hap file).

(2) Fixed distances: it is now possible to fix the distance between a pair of adjacent loci, for the purpose of likelihood calculations, with the options **fixed** and **chrompic**.

(3) The .ord file can optionally be used to prescreen locus orders generated by **all** and **flips*n***, to reduce the number of orders for which likelihoods need be calculated. (Previously the .ord file was only used by the mapbuilding options). The parameter use_ord_file in the .par file controls the use of the .ord file with these options.

(4) **twopoint**: it is now possible to divide the loci into two different groups, and generate LOD tables only for pairs consisting of one locus from each group (this makes it possible, for example, to conveniently generate LOD tables between "new" loci and "old" loci). The parameter PUK_LIKE_TOL is now used as a LOD cutoff: tables are displayed only for locus pairs whose LOD exceeds PUK_LIKE_TOL.

(5) **chrompic**: chromosomes are now labelled by individual ID # (not position within .gen file); phase designations are now always consistent across pedigrees (not merely across chromosomes) even in the phase unknown case; the relative probabilities for the best phase choice and the next most likely alternative are given; the phase designations now distinguish phase known from phase unknown.

(6) a new option, **merge**, makes it easy to merge two .gen files having overlapping sets of families and/or loci.

Other changes in v. 2.4, include: family IDs are now allowed to be arbitrary character strings (not merely integers as in previous versions); the format of the .par file has been changed to make it easier to use; the orders displayed by **all** are sorted by decreasing $\log_{10}$likelihood; output of **flips*n*** is easier to read (the "flipped" loci in each order are highlighted).

BIBLIOGRAPHY

Barker D, Green P, Knowlton R, Schumm J, Lander E, Oliphant A, Willard H, Akots G, Brown V, Gravius T, Helms C, Nelson C, Parker C, Rediker K, Watt D, Weiffenbach B, Donis-Keller H (1987): Genetic linkage map of human chromosome 7 with 63 DNA markers. *Proc. Natl. Acad. Sci. USA* 84: 8006-8010.

Donis-Keller H, Green P, Helms C, Cartinhour S, Weiffenbach B, Stephens K, Keith T, Bowden D, Smith D, Lander E, Botstein D, Akots G, Rediker K, Gravius T, Brown V, Rising M, Parker C, Powers J, Watt D, Kauffman E, Bricker A, Phipps P, Muller-Kahle H, Fulton T, Ng S, Schumm J, Braman J, Knowlton R, Barker D, Crooks S, Lincoln S, Daly M, Abrahamson J (1987): A genetic linkage map of the human genome. *Cell* 51, 319-337.

Goldgar D, Green P, Parry D, Mulvihill J (1989): Multipoint linkage analysis in Neurofibromatosis Type I: An international collaboration. *Am J Human Genetics* 44: 6-12.

Green P (1988): Rapid construction of multilocus genetic linkage maps. I. Maximum likelihood estimation. (draft manuscript).

Kernighan B, Ritchie D (1978): *The C programming language*. Prentice-Hall.

Lander E, Green P (1987): Construction of multilocus genetic linkage maps in humans. *PNAS* 84, 2363-2367.

Ott J (1985): *Analysis of human genetic linkage*. Johns Hopkins University Press.
