# Supplementary material for: The effect of parasite infection on the recombination rate of the mosquito Aedes aegypti
Source: PLoS One. 2018 Oct 9;13(10):e0203481. doi: 10.1371/journal.pone.0203481 (PMC6177114; doi:10.1371/journal.pone.0203481)
Supplement: S2 File — (PDF) [file pone.0203481.s002.pdf]

Exercise 2.1

Linkage analysis

1. There are several mutant eye colours seen in *Drosophila,* one of which is purple, while another mutation affects the length of the wings: vestigial giving a 'scrunched up' look. True-breeding wild red-eyed flies are $\boldsymbol{pr^+pr^+}$ and flies with purple eyes are ***prpr***. Pure breeding, normal-winged flies (wild type) are $\boldsymbol{vg^+vg^+}$ and vestigial-winged flies are ***vgvg***. The wild type is dominant
   Early last century, Morgan and his co-workers carried out many experiments with *Drosophila*. From one of these experiments involving the testcrossing of $\boldsymbol{pr^+pr\ vg^+vg}$ females to ***prpr vgvg*** males the following progeny were scored.

Phenotype	From female gametes	Observed
wild type	$pr^+\ vg^+$	157
normal, vestigial wing	$pr^+\ vg$	965
purple, normal	$pr\ vg^+$	1,067
purple, vestigial	*pr vg*	146
		2,335

– What does this tell us about the two genes?

– Identify the recombinant phenotypes in the progeny.

– Estimate the map distance between the two genes.

– What are the genotypes of the homozygous parental flies of the F_1 females used in the testcrosses? Are the alleles in the parents in coupling phase or repulsion phase?

2. Verify that the LOD score for the 20 progeny example with no prior assumption about phase, should be equal to 2.9 for r = 0.10.

3. Run CRI-map for the 20 progeny example. Verify LOD score, estimated map distance, number of informative progeny.

4. Run CRI-map for the larger example

A short primer how to use CRI-MAP:

CRI-MAP is distributed as source code in the language C and can be obtained by contacting the author at:

Phil Green
Dept. of Genetics
Box 8232
Washington University School of Medicine St. Louis, MO 63110
(314) 362-5192
Fax: (314) 362-4137
email: pg@genome.wustl.edu

Purpose (from the Documentation):
The main purpose of CRI-MAP is to allow rapid, largely automated construction of multilocus linkage maps (and facilitate the attendant tasks of assessing support relative to alternative locus orders, generating LOD tables, and detecting data errors).

Getting Started:

A. You must provide a *.gen file which contains the raw genotype data. The file for example XX should look like:

```
------------------------------- chr1.gen --------------------------------
1                           # of families
2                           # of loci
LOCM   LOCN                 locus name 1, locus name 2, …
FAM1                        family ID
4                           # of members
1   0   0   1               For each member in family: ID, mother's ID, father's
ID, sex
    1   2   1   2           (sex 0 if female, 1 if male, 2 if unknown),
2   0   0   0               locus1 allel1, locus1 allele2, locus2 allele1, locus2
allel2,..
    1   1   1   1
3   1   2   1
    1   1   1   1
----------------------------------------------------------------------
```

B. Now run the prepare option first in order to create the other files required by the program. The command format for running the program is

```
>>    crimap 1 prepare
```

CRI-MAP will then print the default options and ask you if you want to change any of these values:

>> Do you wish to change any of these values? (y/n)

Answer <y> to change the value of PUK_LIKE_TOL from 3.000 to 0.0. This forces CRI-MAP to print out LOD score value smaller than the default value ≥ 3.0
Answer the next question by typing <n> until the program prints:

>> The crimap options are:
>> [1] build [2] instant [3] quick [4] fixed
>> [5] flips [6] all [7] twopoint [8] chrompic
>> Enter the number of the option you will be running next:

Enter <7>, and answer the next questions by <y>.

C. After CRI-MAP have created the parameter file for your analysis, you should type
>> crimap 1 twopoint

An online documentation of CRI-MAP can be found on the web at
http://linkage.rockefeller.edu/
